# Synthesis and crystal structures of (2*E*)-1,4-bis­(4-chloro­phen­yl)but-2-ene-1,4-dione and (2*E*)-1,4-bis­(4-bromo­phen­yl)but-2-ene-1,4-dione

**DOI:** 10.1107/S205698901800230X

**Published:** 2018-02-13

**Authors:** Dominika N. Lastovickova, John J. La Scala, Rosario C. Sausa

**Affiliations:** a US Army Research Laboratory, RDRL-WMM-G, Aberdeen Proving Ground, MD 21005, USA; b US Army Research Laboratory, RDRL-WMM-B, Aberdeen Proving Ground, MD 21005, USA

**Keywords:** crystal structure, synthesis, 1,4-enedione moiety, bis-halo-enedione, NMR

## Abstract

The mol­ecular structure of (2*E*)-1,4-bis­(4-chloro­phen­yl)but-2-ene-1,4-dione [C_16_H_10_Cl_2_O_2_, (**1**)] is composed of two *p*-chlorophenyl rings, each bonded on opposite ends to a near planar 1,4-*trans* enedione moiety [–C(=O)—CH=CH—(C=O)–] [r.m.s. deviation = 0.003 (1) Å]. (2*E*)-1,4-Bis(4-bromo­phen­yl)but-2-ene-1,4-dione [C_16_H_10_Br_2_O_2_, (**2**)] has a similar structure to (**1**), but with two *p*-bromophenyl rings and a less planar enedione group [r.m.s. deviation = 0.011 (1) Å]. Both mol­ecules sit on a center of inversion, thus *Z*′ = 0.5. The dihedral angles between the ring and the enedione group are 16.61 (8) and 15.58 (11)° for (**1**) and (**2**), respectively. In the crystal, mol­ecules of (**1**) exhibit C—Cl⋯Cl type I inter­actions, whereas mol­ecules of (**2**) present C—Br⋯Br type II inter­actions. van der Waals-type inter­actions contribute to the packing of both mol­ecules, and the packing reveals face-to-face ring stacking with similar inter­planar distances of approximately 3.53 Å.

## Abstract 2

The mol­ecular structure of (2*E*)-1,4-bis­(4-chloro­phen­yl)but-2-ene-1,4-dione (**1**) is composed of two *p*-chloro phenyl rings, each bonded on opposite ends to a near planar 1,4-*trans* enedione moiety [–C(= O)—CH=CH—(C=O)–]. (2*E*)-1,4-Bis(4-bromo­phen­yl)but-2-ene-1,4-dione (**2**) has a similar structure to (**1**), but with two *p*-bromo phenyl rings and a less planar enedione group. In the crystal, mol­ecules of (**1**) exhibit C—Cl⋯Cl type I inter­actions, whereas mol­ecules of (**2**) present C–Br⋯Br type II inter­actions.

## Chemical context   

The 1,4-enedione moiety [–C(=O)—CH=CH—(C=O)–] occurs in many natural and bioactive compounds, including steroids, anti­biotics, and anti­tumor agents (Koft & Smith, 1982 ▸; Ismail *et al.*, 1996 ▸; Connolly & Hill, 2010 ▸; Fouad *et al.*, 2006 ▸; Yang *et al.*, 2013 ▸). Its multifunctionality and versatility make it an excellent building block for novel material syntheses. In certain mol­ecules, the facile and reversible *E*/*Z* isomerization of the enedione groups enables them to perform as optical pH and fluorescent sensors (Li *et al.*, 2017 ▸). The title compounds (2*E*)-1,4-bis­(4-chloro­phen­yl)but-2-ene-1,4-dione (**1**) and (2*E*)-1,4-bis­(4-bromo­phen­yl)but-2-ene-1,4-dione (**2**) exhibit two *p*-halogen phenyl rings, each bonded on opposite ends of the enedione group. We have synthesized these compounds in our laboratory as precursors to 4,4′-(furan-2,5-di­yl)dibenzaldehyde cross-linkers. The reduction of the title compounds yields the saturated 1,4-diketones that, under Paal–Knorr reaction conditions, can undergo cyclization to produce the corresponding furans (Sauer *et al.*, 2017 ▸). The aryl halides can be subsequently replaced with formyl groups using the Bouveault aldehyde synthesis to generate the targeted 4,4′-(furan-2,5-di­yl)dibenzaldehyde cross-linkers, which can be potentially used for non-toxic, iso­cyanate-free synthesis of polyurethanes.
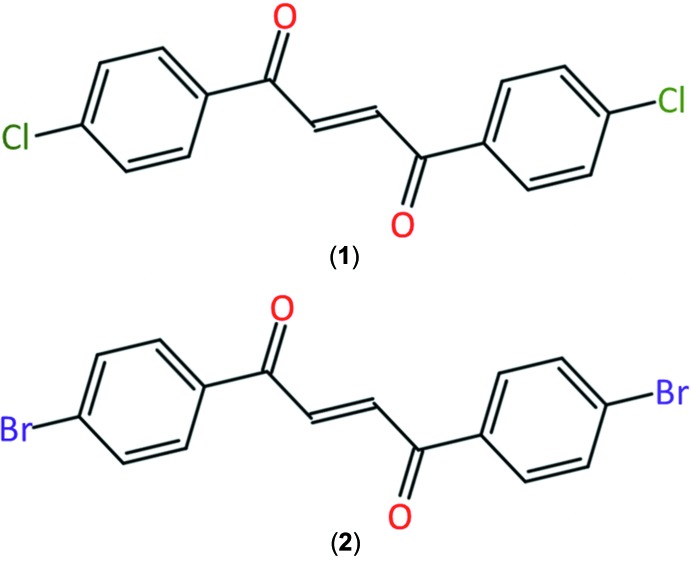


## Structural commentary   

**Figure 1:**
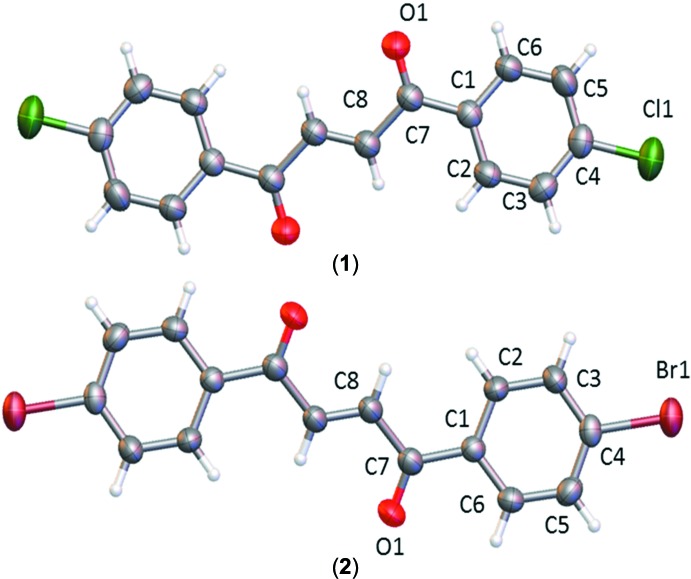
Mol­ecular conformation and atom-numbering scheme of (**1**) (top) and (**2**) (bottom). The non-labeled atoms are generated by symmetry operation (−*x*, 1 − *y*, 1 − *z*) for (**1**) and (1 − *x*, −*y*,1 − *z*) for (**2**). Non-hydrogen atoms are shown as 50% probability displacement ellipsoids.

**Figure 2:**
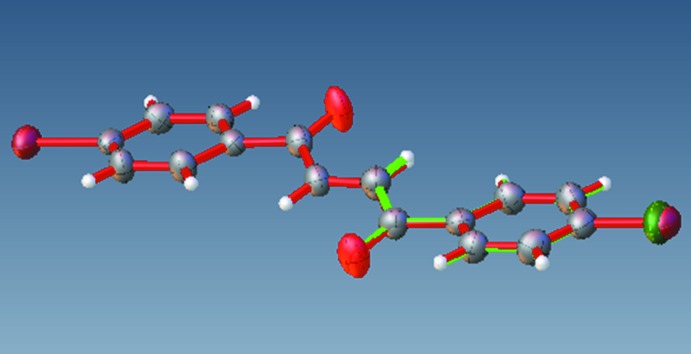
Superimposition of structure (**1**) (green) onto the inverted structure of (**2**) (red). Only the asymmetric unit of (**1**) is presented for clarity.

The title compounds exhibit mol­ecular structures typical of biphenyl enedione compounds (Rabinovich *et al.*, 1970 ▸; Xu *et al.*, 2013 ▸; Li *et al.*, 2014 ▸). Bond lengths and angles are in the usual ranges. Fig. 1 ▸ shows that the mol­ecules sit on centers of inversion and that the enedione groups adopt a *trans*, near planar configuration [r.m.s deviations = 0.003 (1) and 0.011 (1) Å for (**1**) and (**2**), respectively]. In mol­ecule (**1**), the carbonyl group is twisted slightly out of the chloro­phenyl plane, as evidenced by the torsion angles C6—C1—C7—O1 [−15.6 (3)°] and C2—C1—C7—O1 [163.9 (2)°]. Mol­ecule (**2**) shows a similar conformation with torsion angles of 14.5 (4) and −164.7 (3)° for the corresponding atoms of the inverted asymmetric unit (−*x* + 1, −*y*, −*z* + 1). The chloro­phenyl ring planes form a dihedral angle of 16.61 (8)° with respect to the enedione plane (O1–C7–C8–C8′–C7′–O1′) in (**1**), whereas the bromo­phenyl ring planes form a dihedral angle of 15.58 (11)° relative to the enedione plane in (**2**). Both mol­ecules exhibit a pair of short intra­molecular H⋯H contacts [(**1**): H2⋯H8 = H2^i^⋯H8^i^ = 2.127 (2) Å; symmetry code (i): −*x*, 1 − *y*, 1 − *z*; and (**2**): H2⋯H8 = H2^ii^⋯H8^ii^ = 2.113 (3) Å; symmetry code: (ii) 1 − *x*, −y, 1 − *z*], possibly resulting from steric compression of the large phenyl halogen groups. A best fit of all symmetry independent atoms of both mol­ecules (see Fig. 2 ▸) yields an r.m.s. deviation of 0.05 Å.

## Supra­molecular features   

**Figure 3:**
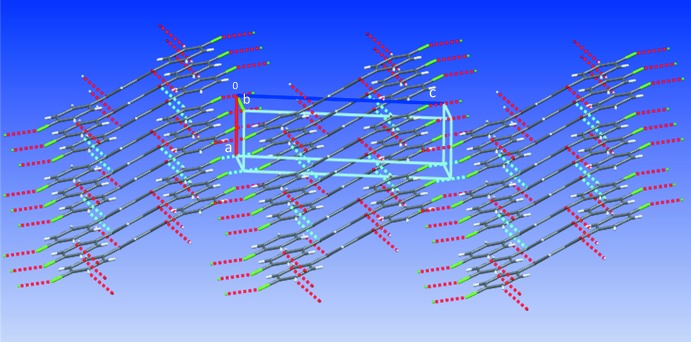
Crystal packing of (**1**) along the vicinity of the *a* axis. Dashed lines depict Cl1⋯Cl1^i^ and O1⋯H3^ii^ inter­actions [symmetry codes: (i) 2 − *x*, −*y*, −*z*; (ii) −1 + *x*, 1 + *y*, *z*].

**Figure 4:**
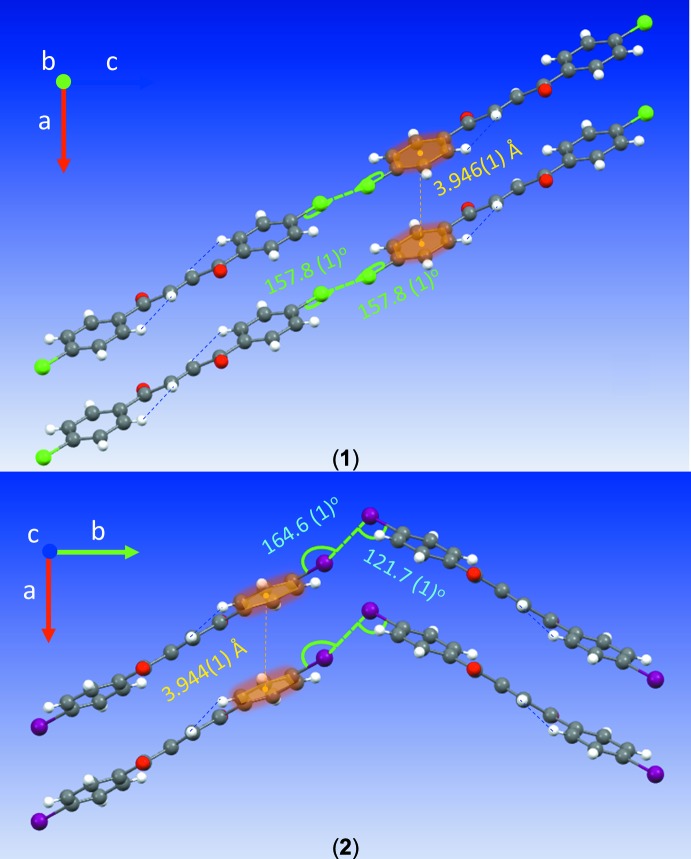
Mol­ecular conformations of (**1**) and (**2**) viewed along the *b* and *c* axes, respectively, showing type I and II halogen inter­actions, centroid-to-centroid distances, and short intra­molecular H⋯H inter­actions.

**Figure 5:**
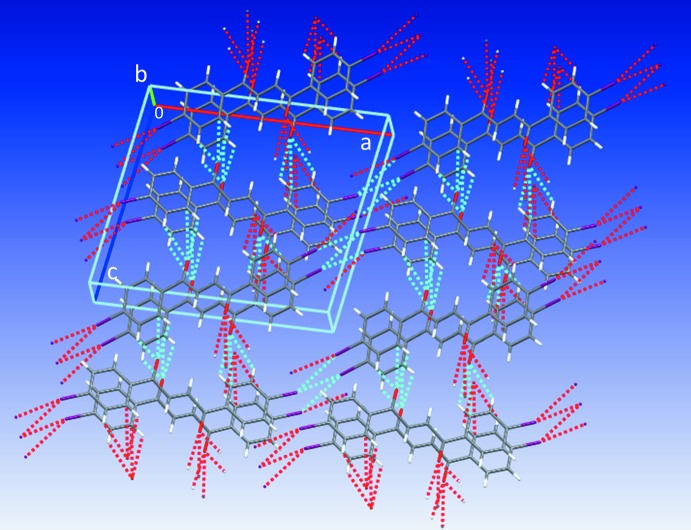
Crystal packing of (**2**) along the *b* axis. Dashed blue lines represent bifurcated Br1⋯Br1^iv,v^ inter­actions [symmetry codes: (iv) −*x*, −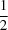 + *y*, 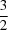 − *z*; (v) −*x*, 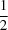 + *y*, 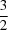 − *z*] and trifurcated interactions involving the O1 atoms.

Contacts between the O atoms and H atoms of adjacent mol­ecules [O1⋯H3^i^ = 3.329 (2) Å; symmetry code: (i) −1 + *x*, 1 + *y*, *z*] and between the Cl atoms and Cl atoms of adjacent mol­ecules [Cl1⋯Cl1^ii^ = 3.3841 (1) Å; symmetry code: (ii) 2 − *x*, −*y*, −*z*] contribute to the inter­molecular inter­actions of (**1**) (see Fig. 3 ▸). The short Cl⋯Cl distances are approximately 0.3 Å shorter than double the Cl van der Waals radius of 3.64 Å (Alvarez, 2013 ▸). The mol­ecules feature type I, C_*w*_—Cl_*x*_⋯Cl_*y*_—C_*z*_ inter­actions, where θ_1_ = angle C_*w*_—Cl_*x*_⋯Cl_*y*_, θ_2_ = angle Cl_*x*_⋯Cl_*y*_—C_*z*_, and |θ_1_ − θ_2_| = 0 (θ_1_ = θ_2_, approximately 157°) (see Fig. 4 ▸), suggesting that the Cl atoms minimize repulsion by inter­facing the neutral regions of their electrostatic potential surfaces (Desiraju & Parthasarathy, 1989 ▸; Mukherjee & Desiraju, 2014 ▸). Unlike (**1**), (**2**) exhibits trifurcated contacts between the O atoms and H and C atoms of adjacent mol­ecules [O1⋯H2^iii^ = 2.616 (2) Å, O1⋯H3^iii^ = 2.711 (2) Å, and O1⋯C2^iii^ = 3.194 (3) Å; symmetry code: (iii) *x*, 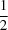 − *y*, 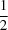 + *z*]. Furthermore, the Br atoms form bifurcated contacts with the Br atoms of adjacent mol­ecules [Br1⋯Br^1v^ = Br1⋯Br1^v^ = 3.662 (1) Å; symmetry codes: (iv) −*x*, −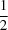 + *y*, 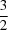 − *z*; (v) −*x*, 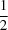 + *y*, 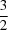 − *z*] (see Fig. 5 ▸). Inspection of the C—Br⋯Br—C angles, reveals that the mol­ecules exhibit type II inter­actions (|θ_1_ − θ_2_| ≥ 30°, where θ_1_ (164.58°) − θ_2_ (121.71°) = 42.87°, suggesting the electrophilic region of one Br atom approaches the nucleophilic region of the companion Br atom, unlike the Cl⋯Cl inter­actions (Mukherjee & Desiraju, 2014 ▸; Tothadi *et al.*, 2013 ▸; Nuzzo *et al.*, 2017 ▸). The chloro­phenyl rings (**1**) are stacked in close proximity along the vicinity of the *a* axis with an inter­planar separation of 3.528 Å [centroid-to-centroid distance = 3.946 (1) Å] (see Figs. 4 ▸ and 5 ▸). Similarly, the bromo phenyl rings of (**2**) stack along the vicinity of the *a* axis with an inter­planar separation of 3.525 Å [centroid-to-centroid distance = 3.994 (1) Å], but in a crisscross-like pattern when viewed along the *c* axis (see Figs. 3 ▸ and 5 ▸). The inter­secting ring planes subtend dihedral angles of 48.09 (6)°.

## Database survey   

A search of the Cambridge Structural Database (CSD web interface; Groom *et al.*, 2016 ▸) and the Crystallography Open Database (Gražulis *et al.*, 2009 ▸) yields the crystal structures of a number of compounds containing the 1,4-enedione moiety. For examples, see Rabinovich *et al.* (1970 ▸), Xu *et al.* (2013 ▸), Li *et al.* (2014 ▸), Deng *et al.* (2012 ▸); Gao *et al.* (2010 ▸), and Wu *et al.* (2011 ▸). The compounds *trans*-1,2-di­phenyl­ethyl­ene (**3**) (Xu *et al.*, 2013 ▸; CCDC 918566, BZOYEY01) and *cis*-1,2-di­chloro­benzoyl­ethyl­ene (**4**) (Rabinovich *et al.*, 1970 ▸; CCDC 112151, CBOZET) merit discussion because the former has a similar structure to the title compounds, whereas the latter is a stereoisomer of (**1**). The title compounds adopt an *E* configuration, similar to (**3**). They contain halogen atoms in the *para* position of the phenyl groups, unlike (**3**), but the rings are nearly planar as are those of (**3**), whose r.m.s value = 0.008 Å. The r.m.s. value, reflecting the planarity of the enedione moiety, in (**1**) is different to that of (**3**) (0.003 *vs* 0.0035 Å), and the value determined for (**2**) (0.011 Å). The dihedral angles between the ring planes of (**1**) and (**2**) are nearly identical to those of (**3**) [16° (average) *vs* 15.7 (1)°]. Unlike (**1**), its diastereomer (**4**) does not exhibit a planar enedione moiety and its near planar chloro­phenyl rings (r.m.s deviation = 0.018 Å) form a dihedral angle of 77.4 (3)° with respect to each other. Superimposition of atom C1 of the *E*/*Z* diastereomers through the C7, Cl1, and O1 atoms yields an r.m.s. deviation of 0.033 Å. The remaining parts of the mol­ecules are twisted from each other, with the planes containing the chloro­phenyl group and adjoining carbonyl group of each stereoisomer forming a dihedral angle of approximately 79°.

## Synthesis and crystallization   

The title compounds were synthesized following a modified literature procedure (Sauer *et al.*, 2017 ▸). The reactions were run ‘neat’ with chloro- or bromo­benzene used in excess and serving also as the reaction solvent. Under a stream of nitro­gen, aluminum chloride (3.6 g, 27 mmol, 2.9 equiv.) was dissolved in chloro- or bromo­benzene (9.0 and 9.3 ml, respectively, 89 mmol, 9.6 equiv.) at room temperature. The reaction mixture was subsequently cooled to 273 K and fumaryl chloride (1.0 ml, 9.3 mmol, 1.0 equiv.) was added dropwise under constant stirring, at which point an instantaneous color change from clear to deep red was observed. The reaction mixture was then heated to 333 K for 2–4 days until fumaryl chloride was no longer detected on a TLC plate (SiO_2_, DCM). At the conclusion of the reaction, the mixture was cooled to room temperature, poured into ice-cold aqueous 1 *M* HCl, and extracted several times with DCM. The combined organic layers were washed with 0.5 *M* NaOH and dried over Na_2_SO_4_, and the volatiles were removed under reduced pressure. The resulting red–brown solid was recrystallized in DCM, further purified with a series of cold DCM washes, and dried under reduced pressure, affording either compound (**1**) (burnt orange solid, 1.5 g, 4.9 mmol, 53% yield) or (**2**) (yellow solid, 1.9 g, 4.8 mmol, 50% yield). Slow evaporation of DCM solutions saturated with either (**1**) or (**2**) yielded single crystals suitable for X-ray diffraction.

NMR spectra were recorded on a Bruker 400 MHz spectrometer. Chemical shifts (δ) are given in ppm and are referenced to tetra­methyl­silane (TMS) using the residual solvent (^1^H: CDCl_3_, 7.26 ppm; ^13^C: CDCl_3_, 77.16 ppm). (**1**): ^1^H NMR (CDCl_3_, 400.13 MHz): δ 7.51 (*d*, *J* = 8.6 Hz, 4H), 7.97 (*s*, 2H), 8.00 (*d*, *J* = 8.6 Hz, 4H) ppm. ^13^C NMR (CDCl_3_, 100.62 MHz): δ 129.48, 130.40, 135.06, 135.31, 140.77, 188.51 ppm. (**2**): ^1^H NMR (CDCl_3_, 400.13 MHz): δ 7.67 (*d*, *J* = 8.6 Hz, 4H), 7.92 (*d*, *J* = 8.6 Hz, 4H), 7.96 (s, 2H) ppm. ^13^C NMR (CDCl_3_, 100.62 MHz): δ 129.53, 130.44, 132.45, 135.03, 135.69, 188.69 ppm.

## Refinement   

Crystal data, data collection and structure refinement details are summarized in Table 1 ▸. The hydrogen atoms of both compounds were refined using a riding model with C—H = 0.93 Å and *U*_iso_(H) = 1.2*U*_eq_(C).

## Supplementary Material

Crystal structure: contains datablock(s) 1, 2. DOI: 10.1107/S205698901800230X/zl2724sup1.cif

Structure factors: contains datablock(s) 1. DOI: 10.1107/S205698901800230X/zl27241sup4.hkl

Structure factors: contains datablock(s) 2. DOI: 10.1107/S205698901800230X/zl27242sup5.hkl

Supporting information file. DOI: 10.1107/S205698901800230X/zl27241sup4.cml

Supporting information file. DOI: 10.1107/S205698901800230X/zl27242sup5.cml

CCDC references: 1822698, 1822697

Additional supporting information:  crystallographic information; 3D view; checkCIF report

## Figures and Tables

**Table 1:** Experimental details

	(**1**)	(**2**)
Crystal data		
Chemical formula	C_16_H_10_Cl_2_O_2_	C_16_H_10_Br_2_O_2_
*M*_r_	305.14	394.06
Crystal system, space group	Triclinic, *P* 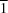	Monoclinic, *P*2_1_/*c*
Temperature (K)	298	298
*a*, *b*, *c* (Å)	3.9455 (3), 6.0809 (5), 14.6836 (11)	14.4391 (7), 3.9937 (2), 12.7244 (7)
α, β, γ (°)	82.653 (6), 88.638 (6), 84.601 (7)	90, 97.827 (5), 90
*V* (Å^3^)	347.82 (5)	726.92 (7)
*Z*	1	2
Radiation type	Mo *K*α	Mo *K*α
μ (mm^−1^)	0.46	5.57
Crystal size (mm)	0.34 × 0.22 × 0.15	0.35 × 0.14 × 0.12

Data collection		
Diffractometer	Agilent SuperNova, Dualflex, EosS2	Agilent SuperNova, Dualflex, EosS2
Absorption correction	Multi-scan (*CrysAlis PRO*; Bourhis *et al.*, 2015 ▸)	Multi-scan (*CrysAlis PRO*; Bourhis *et al.*, 2015 ▸)
*T*_min_, *T*_max_	0.928, 1.000	0.370, 1.000
No. of measured, independent and observed [*I* > 2σ(*I*)] reflections	5641, 1416, 1256	6231, 1470, 1228
*R*_int_	0.023	0.031
(sin θ/λ)_max_ (Å^−1^)	0.625	0.625

Refinement		
*R*[*F* ^2^ > 2σ(*F* ^2^)], *wR*(*F* ^2^), *S*	0.048, 0.104, 1.16	0.029, 0.065, 1.08
No. of reflections	1416	1470
No. of parameters	91	92
H-atom treatment	H-atom parameters constrained	H-atom parameters constrained
Δρ_max_, Δρ_min_ (e Å^−3^)	0.18, −0.20	0.36, −0.43

Computer programs: *CrysAlis PRO* (Rigaku OD, 2015 ▸), *SHELXT* (Sheldrick, 2015*a* ▸), *SHELXL* (Sheldrick, 2015*b* ▸), *OLEX2* (Dolomanov *et al.*, 2009 ▸) and *Mercury* (Macrae *et al.*, 2008 ▸).

## supplementary crystallographic information

### (2E)-1,4-Bis(4-chlorophenyl)but-2-ene-1,4-dione (1) Crystal data

**Table:**

C_16_H_10_Cl_2_O_2_	*Z* = 1
*M**_r_* = 305.14	*F*(000) = 156
Triclinic, *P*1	*D*_x_ = 1.457 Mg m^−^^3^
*a* = 3.9455 (3) Å	Mo *K*α radiation, λ = 0.71073 Å
*b* = 6.0809 (5) Å	Cell parameters from 2009 reflections
*c* = 14.6836 (11) Å	θ = 2.8–26.3°
α = 82.653 (6)°	µ = 0.46 mm^−^^1^
β = 88.638 (6)°	*T* = 298 K
γ = 84.601 (7)°	Irregular, orange
*V* = 347.82 (5) Å^3^	0.34 × 0.22 × 0.15 mm

### (2E)-1,4-Bis(4-chlorophenyl)but-2-ene-1,4-dione (1) Data collection

**Table:**

Agilent SuperNova, Dualflex, EosS2 diffractometer	1256 reflections with *I* > 2σ(*I*)
Detector resolution: 8.0945 pixels mm^-1^	*R*_int_ = 0.023
ω scans	θ_max_ = 26.4°, θ_min_ = 2.8°
Absorption correction: multi-scan (CrysAlisPro; Bourhis *et al.*, 2015)	*h* = −4→4
*T*_min_ = 0.928, *T*_max_ = 1.000	*k* = −7→7
5641 measured reflections	*l* = −18→18
1416 independent reflections	

### (2E)-1,4-Bis(4-chlorophenyl)but-2-ene-1,4-dione (1) Refinement

**Table:**

Refinement on *F*^2^	0 restraints
Least-squares matrix: full	Hydrogen site location: inferred from neighbouring sites
*R*[*F*^2^ > 2σ(*F*^2^)] = 0.048	H-atom parameters constrained
*wR*(*F*^2^) = 0.104	*w* = 1/[σ^2^(*F*_o_^2^) + (0.0335*P*)^2^ + 0.118*P*] where *P* = (*F*_o_^2^ + 2*F*_c_^2^)/3
*S* = 1.16	(Δ/σ)_max_ < 0.001
1416 reflections	Δρ_max_ = 0.18 e Å^−^^3^
91 parameters	Δρ_min_ = −0.19 e Å^−^^3^

### (2E)-1,4-Bis(4-chlorophenyl)but-2-ene-1,4-dione (1) Special details

**Table:**

Geometry. All esds (except the esd in the dihedral angle between two l.s. planes) are estimated using the full covariance matrix. The cell esds are taken into account individually in the estimation of esds in distances, angles and torsion angles; correlations between esds in cell parameters are only used when they are defined by crystal symmetry. An approximate (isotropic) treatment of cell esds is used for estimating esds involving l.s. planes.

### (2E)-1,4-Bis(4-chlorophenyl)but-2-ene-1,4-dione (1) Fractional atomic coordinates and isotropic or equivalent isotropic displacement parameters (Å^2^)

**Table:**

	*x*	*y*	*z*	*U*_iso_*/*U*_eq_	
C1	0.3800 (5)	0.4949 (3)	0.30832 (13)	0.0411 (4)	
C2	0.5146 (5)	0.2732 (3)	0.31865 (14)	0.0482 (5)	
H2	0.493021	0.185559	0.374837	0.058*	
C3	0.6788 (6)	0.1826 (4)	0.24675 (15)	0.0529 (5)	
H3	0.771626	0.035173	0.254438	0.063*	
C4	0.7048 (5)	0.3111 (4)	0.16369 (14)	0.0500 (5)	
C5	0.5749 (6)	0.5314 (4)	0.15096 (15)	0.0559 (6)	
H5	0.595485	0.617285	0.094315	0.067*	
C6	0.4149 (5)	0.6214 (3)	0.22341 (14)	0.0495 (5)	
H6	0.327970	0.770037	0.215556	0.059*	
C7	0.2047 (5)	0.6004 (3)	0.38415 (14)	0.0459 (5)	
C8	0.0820 (5)	0.4582 (3)	0.46605 (13)	0.0452 (5)	
H8	0.123290	0.304322	0.469025	0.054*	
Cl1	0.90353 (18)	0.19244 (12)	0.07252 (4)	0.0768 (3)	
O1	0.1558 (5)	0.8021 (2)	0.38003 (11)	0.0699 (5)	

### (2E)-1,4-Bis(4-chlorophenyl)but-2-ene-1,4-dione (1) Atomic displacement parameters (Å^2^)

**Table:**

	*U*^11^	*U*^22^	*U*^33^	*U*^12^	*U*^13^	*U*^23^
C1	0.0389 (10)	0.0429 (10)	0.0419 (10)	−0.0081 (8)	−0.0005 (8)	−0.0038 (8)
C2	0.0565 (13)	0.0421 (11)	0.0450 (11)	−0.0066 (9)	0.0017 (9)	−0.0003 (9)
C3	0.0558 (13)	0.0447 (11)	0.0587 (13)	−0.0013 (10)	−0.0009 (10)	−0.0115 (10)
C4	0.0463 (12)	0.0595 (13)	0.0471 (12)	−0.0076 (10)	0.0021 (9)	−0.0164 (10)
C5	0.0616 (14)	0.0622 (13)	0.0413 (11)	−0.0054 (11)	0.0049 (10)	0.0017 (10)
C6	0.0539 (12)	0.0437 (11)	0.0481 (12)	−0.0008 (9)	0.0031 (9)	0.0017 (9)
C7	0.0480 (11)	0.0440 (11)	0.0450 (11)	−0.0066 (9)	0.0022 (9)	−0.0017 (8)
C8	0.0477 (12)	0.0431 (10)	0.0440 (11)	−0.0046 (9)	0.0015 (8)	−0.0023 (8)
Cl1	0.0799 (5)	0.0926 (5)	0.0621 (4)	−0.0021 (4)	0.0148 (3)	−0.0326 (3)
O1	0.1034 (14)	0.0410 (8)	0.0617 (10)	−0.0006 (8)	0.0245 (9)	−0.0015 (7)

### (2E)-1,4-Bis(4-chlorophenyl)but-2-ene-1,4-dione (1) Geometric parameters (Å, º)

**Table:**

C1—C2	1.392 (3)	C4—Cl1	1.737 (2)
C1—C6	1.390 (3)	C5—H5	0.9300
C1—C7	1.481 (3)	C5—C6	1.372 (3)
C2—H2	0.9300	C6—H6	0.9300
C2—C3	1.374 (3)	C7—C8	1.488 (3)
C3—H3	0.9300	C7—O1	1.218 (2)
C3—C4	1.369 (3)	C8—C8^i^	1.307 (4)
C4—C5	1.379 (3)	C8—H8	0.9300
			
C2—C1—C7	122.61 (18)	C4—C5—H5	120.6
C6—C1—C2	118.30 (19)	C6—C5—C4	118.8 (2)
C6—C1—C7	119.08 (18)	C6—C5—H5	120.6
C1—C2—H2	119.6	C1—C6—H6	119.3
C3—C2—C1	120.72 (19)	C5—C6—C1	121.3 (2)
C3—C2—H2	119.6	C5—C6—H6	119.3
C2—C3—H3	120.3	C1—C7—C8	119.63 (17)
C4—C3—C2	119.4 (2)	O1—C7—C1	120.95 (18)
C4—C3—H3	120.3	O1—C7—C8	119.41 (19)
C3—C4—C5	121.4 (2)	C7—C8—H8	118.8
C3—C4—Cl1	118.98 (17)	C8^i^—C8—C7	122.3 (2)
C5—C4—Cl1	119.60 (17)	C8^i^—C8—H8	118.8
			
C1—C2—C3—C4	−1.1 (3)	C4—C5—C6—C1	−0.4 (3)
C1—C7—C8—C8^i^	−178.2 (2)	C6—C1—C2—C3	0.3 (3)
C2—C1—C6—C5	0.5 (3)	C6—C1—C7—C8	163.48 (18)
C2—C1—C7—C8	−17.0 (3)	C6—C1—C7—O1	−15.6 (3)
C2—C1—C7—O1	163.9 (2)	C7—C1—C2—C3	−179.24 (19)
C2—C3—C4—C5	1.2 (3)	C7—C1—C6—C5	−179.94 (19)
C2—C3—C4—Cl1	−178.37 (16)	Cl1—C4—C5—C6	179.15 (16)
C3—C4—C5—C6	−0.4 (3)	O1—C7—C8—C8^i^	0.9 (4)

Symmetry code: (i) −*x*, −*y*+1, −*z*+1.

### (2E)-1,4-Bis(4-bromophenyl)but-2-ene-1,4-dione (2) Crystal data

**Table:**

C_16_H_10_Br_2_O_2_	*F*(000) = 384
*M**_r_* = 394.06	*D*_x_ = 1.800 Mg m^−^^3^
Monoclinic, *P*2_1_/*c*	Mo *K*α radiation, λ = 0.71073 Å
*a* = 14.4391 (7) Å	Cell parameters from 2527 reflections
*b* = 3.9937 (2) Å	θ = 2.9–26.2°
*c* = 12.7244 (7) Å	µ = 5.57 mm^−^^1^
β = 97.827 (5)°	*T* = 298 K
*V* = 726.92 (7) Å^3^	Irregular, yellow
*Z* = 2	0.35 × 0.14 × 0.12 mm

### (2E)-1,4-Bis(4-bromophenyl)but-2-ene-1,4-dione (2) Data collection

**Table:**

Agilent SuperNova, Dualflex, EosS2 diffractometer	1228 reflections with *I* > 2σ(*I*)
Detector resolution: 8.0945 pixels mm^-1^	*R*_int_ = 0.031
ω scans	θ_max_ = 26.4°, θ_min_ = 2.9°
Absorption correction: multi-scan (CrysAlisPro; Bourhis *et al.*, 2015)	*h* = −18→18
*T*_min_ = 0.370, *T*_max_ = 1.000	*k* = −4→4
6231 measured reflections	*l* = −15→15
1470 independent reflections	

### (2E)-1,4-Bis(4-bromophenyl)but-2-ene-1,4-dione (2) Refinement

**Table:**

Refinement on *F*^2^	Hydrogen site location: inferred from neighbouring sites
Least-squares matrix: full	H-atom parameters constrained
*R*[*F*^2^ > 2σ(*F*^2^)] = 0.029	*w* = 1/[σ^2^(*F*_o_^2^) + (0.0234*P*)^2^ + 0.4382*P*] where *P* = (*F*_o_^2^ + 2*F*_c_^2^)/3
*wR*(*F*^2^) = 0.065	(Δ/σ)_max_ < 0.001
*S* = 1.08	Δρ_max_ = 0.36 e Å^−^^3^
1470 reflections	Δρ_min_ = −0.43 e Å^−^^3^
92 parameters	Extinction correction: SHELXL-2016/4 (Sheldrick, 2015b), Fc^*^=kFc[1+0.001xFc^2^λ^3^/sin(2θ)]^-1/4^
0 restraints	Extinction coefficient: 0.0047 (8)

### (2E)-1,4-Bis(4-bromophenyl)but-2-ene-1,4-dione (2) Special details

**Table:**

Geometry. All esds (except the esd in the dihedral angle between two l.s. planes) are estimated using the full covariance matrix. The cell esds are taken into account individually in the estimation of esds in distances, angles and torsion angles; correlations between esds in cell parameters are only used when they are defined by crystal symmetry. An approximate (isotropic) treatment of cell esds is used for estimating esds involving l.s. planes.

### (2E)-1,4-Bis(4-bromophenyl)but-2-ene-1,4-dione (2) Fractional atomic coordinates and isotropic or equivalent isotropic displacement parameters (Å^2^)

**Table:**

	*x*	*y*	*z*	*U*_iso_*/*U*_eq_	
Br1	0.06905 (2)	0.86344 (8)	0.66837 (3)	0.05337 (16)	
C1	0.31059 (19)	0.3876 (7)	0.5110 (2)	0.0355 (6)	
C2	0.3212 (2)	0.4543 (8)	0.6188 (2)	0.0444 (7)	
H2	0.377230	0.400535	0.660470	0.053*	
C3	0.2503 (2)	0.5990 (8)	0.6653 (2)	0.0471 (8)	
H3	0.258517	0.646056	0.737543	0.057*	
C4	0.1673 (2)	0.6726 (6)	0.6034 (2)	0.0391 (7)	
C5	0.1538 (2)	0.6073 (7)	0.4962 (2)	0.0463 (7)	
H5	0.097052	0.657253	0.455312	0.056*	
C6	0.2258 (2)	0.4666 (8)	0.4507 (2)	0.0422 (7)	
H6	0.217497	0.423632	0.378154	0.051*	
C7	0.3859 (2)	0.2399 (8)	0.4570 (2)	0.0418 (7)	
C8	0.4672 (2)	0.0760 (7)	0.5218 (2)	0.0410 (7)	
H8	0.471561	0.081856	0.595399	0.049*	
O1	0.38162 (17)	0.2478 (7)	0.36132 (17)	0.0665 (7)	

### (2E)-1,4-Bis(4-bromophenyl)but-2-ene-1,4-dione (2) Atomic displacement parameters (Å^2^)

**Table:**

	*U*^11^	*U*^22^	*U*^33^	*U*^12^	*U*^13^	*U*^23^
Br1	0.0433 (2)	0.0504 (2)	0.0706 (3)	0.00725 (15)	0.02309 (16)	0.00106 (17)
C1	0.0354 (15)	0.0380 (14)	0.0334 (15)	−0.0010 (12)	0.0057 (12)	0.0011 (12)
C2	0.0380 (17)	0.0601 (19)	0.0340 (16)	0.0088 (14)	0.0012 (13)	−0.0001 (14)
C3	0.0465 (18)	0.0598 (19)	0.0359 (16)	0.0119 (15)	0.0082 (13)	−0.0026 (15)
C4	0.0371 (16)	0.0344 (15)	0.0487 (18)	0.0009 (12)	0.0158 (13)	0.0028 (13)
C5	0.0371 (16)	0.0514 (18)	0.0487 (18)	0.0054 (14)	0.0002 (13)	0.0034 (15)
C6	0.0439 (17)	0.0491 (17)	0.0328 (15)	0.0031 (14)	0.0030 (13)	−0.0006 (13)
C7	0.0395 (16)	0.0480 (16)	0.0382 (17)	−0.0016 (13)	0.0060 (13)	−0.0057 (13)
C8	0.0364 (16)	0.0514 (18)	0.0357 (15)	−0.0005 (13)	0.0060 (12)	−0.0064 (14)
O1	0.0591 (15)	0.109 (2)	0.0322 (12)	0.0232 (14)	0.0078 (11)	−0.0071 (12)

### (2E)-1,4-Bis(4-bromophenyl)but-2-ene-1,4-dione (2) Geometric parameters (Å, º)

**Table:**

Br1—C4	1.896 (3)	C4—C5	1.376 (4)
C1—C2	1.385 (4)	C5—H5	0.9300
C1—C6	1.390 (4)	C5—C6	1.377 (4)
C1—C7	1.485 (4)	C6—H6	0.9300
C2—H2	0.9300	C7—C8	1.492 (4)
C2—C3	1.378 (4)	C7—O1	1.211 (3)
C3—H3	0.9300	C8—C8^i^	1.310 (5)
C3—C4	1.374 (4)	C8—H8	0.9300
			
C2—C1—C6	118.3 (3)	C4—C5—H5	120.6
C2—C1—C7	123.1 (3)	C4—C5—C6	118.8 (3)
C6—C1—C7	118.7 (3)	C6—C5—H5	120.6
C1—C2—H2	119.4	C1—C6—H6	119.4
C3—C2—C1	121.2 (3)	C5—C6—C1	121.3 (3)
C3—C2—H2	119.4	C5—C6—H6	119.4
C2—C3—H3	120.5	C1—C7—C8	119.3 (2)
C4—C3—C2	119.0 (3)	O1—C7—C1	121.0 (3)
C4—C3—H3	120.5	O1—C7—C8	119.7 (3)
C3—C4—Br1	118.8 (2)	C7—C8—H8	119.0
C3—C4—C5	121.5 (3)	C8^i^—C8—C7	121.9 (4)
C5—C4—Br1	119.7 (2)	C8^i^—C8—H8	119.0
			
Br1—C4—C5—C6	−179.6 (2)	C3—C4—C5—C6	−0.4 (5)
C1—C2—C3—C4	1.0 (5)	C4—C5—C6—C1	0.6 (5)
C1—C7—C8—C8^i^	175.8 (3)	C6—C1—C2—C3	−0.8 (5)
C2—C1—C6—C5	−0.1 (4)	C6—C1—C7—C8	−164.8 (3)
C2—C1—C7—C8	16.0 (4)	C6—C1—C7—O1	14.5 (4)
C2—C1—C7—O1	−164.7 (3)	C7—C1—C2—C3	178.4 (3)
C2—C3—C4—Br1	178.8 (2)	C7—C1—C6—C5	−179.3 (3)
C2—C3—C4—C5	−0.5 (5)	O1—C7—C8—C8^i^	−3.5 (6)

Symmetry code: (i) −*x*+1, −*y*, −*z*+1.
